# Regulatory T cells (Tregs) in liver fibrosis

**DOI:** 10.1038/s41420-023-01347-8

**Published:** 2023-02-09

**Authors:** Ke-jia Wu, Qu-fei Qian, Jin-ren Zhou, Dong-lin Sun, Yun-fei Duan, Xi Zhu, Kurt Sartorius, Yun-jie Lu

**Affiliations:** 1 Department of Hepatobiliary and Pancreatic Surgery, The Third Affiliated Hospital of Soochow University, Changzhou, 213000 China; 2 The First Affiliated Hospital of Nanjing Medical University, Nanjing, 210000 China; 3 Department of infectious diseases, the First Peoples’ Hospital of Kunshan, 215300 Kunshan, China; 4 Faculty of Commerce, Law and Management, University of the Witwatersrand, Johannesburg, South Africa; 5 School of Laboratory Medicine and Molecular Sciences, University of Kwazulu-Natal, Durban, South Africa; 6 Africa Hepatopancreatobiliary Cancer Consortium, Mayo Clinic, Jacksonville, USA

**Keywords:** T cells, Lymphoid tissues

## Abstract

The ability of the human liver to both synthesize extracellular matrix(ECM), as well as regulate fibrogenesis, are integral functions to maintaining homoeostasis. Chronic liver injury stimulates fibrogenesis in response to the imbalance between ECM accumulation and fibrosis resolution. Liver disease that induces fibrogenesis is associated with multiple risk factors like hepatitis infection, schistosomiasis, alcohol, certain drugs, toxicants and emerging aetiology like diabetes and obesity. The activation of hepatic stellate cells (HSCs), whose function is to generate and accumulate ECM, is a pivotal event in liver fibrosis. Simultaneously, HSCs selectively promote regulatory T-cells (Tregs) in an interleukin-2–dependent pattern that displays a dual relationship. On the one hand, Tregs can protect HSCs from NK cell attack, while on the other hand, they demonstrate an inhibitory effect on HSCs. This paper reviews the dual role of Tregs in liver fibrogenesis which includes its promotion of immunosuppression, as well as its activation of fibrosis. In particular, the balance between Tregs and the Th17 cell population, which produce interleukin (IL)-17 and IL-22, is explored to demonstrate their key role in maintaining homoeostasis and immunoregulation. The contradictory roles of Tregs in liver fibrosis in different immune microenvironments and molecular pathways need to be better understood if they are to be deployed to manage this disease.

## Introduction

Liver fibrosis is generally considered to be the consequence of ongoing chronic liver injury and a leading pathogenic factor of morbidity and mortality in chronic viral hepatitis and obesity-related fatty liver disease worldwide [1]. Non-resolving liver fibrogenesis, stimulated by a wide range of risk factors, induces the activation of quiescent hepatic stellate cells (HSCs) into myofibroblasts that are the primary extracellular matrix (ECM)-cell type that promotes fibrous protein deposition and scarring [2]. The severity and outcome of fibrogenesis in liver disease can be triggered by a wide range of risk factors including viral infection, toxins, and a range of non-communicable diseases that contribute to a build-up of fatty tissue in the liver. Chronic hepatitis infection, for instance, promotes liver fibrosis that can translate into cirrhosis and the onset of neoplasms (Chronic hepatitis → liver fibrosis → liver cirrhosis → Liver Neoplasms) [3]. In patients suffering from chronic liver injury, progression to the end stage usually takes 20 to 40 years depending on whether both environmental and genetic elements are causal factors [1]. Early-stage fibrosis is more easily resolved while advanced liver fibrosis can result in a range of severe symptoms, such as cirrhosis, liver failure, and portal hypertension [4], When fibrogenesis progresses to decompensated cirrhosis, the survival period of patients is significantly shortened, and therapeutic options are eventually limited to liver transplantation [5].

Fibrogenesis as a result of liver injury involves a complex interactome that includes the interaction of various types of immune cells that respond to chronic inflammation, tissue regeneration, ECM remodelling and fibrogenesis [6]. A hallmark event of liver fibrosis is the activation and expression of HSCs which are regulated by several immune mediators, including Th9, Th22, Tregs, innate lymphoid cells (ILCs), mucosal-associated invariant T cells (MAIT), and γδT cells, along with their associated cytokines [7]. In early-stage fibrosis that is resolved, the immune response contributes to terminating HSC activation and fibrous protein expression, however, in the case of ongoing liver disease, the complicated interplay of these immune mediators often promotes liver fibrosis. A key change in the adaptive immune response is the mobilization of regulatory T(Tregs) cells that play an immunosuppressive role to promote immune self-tolerance and homoeostasis. The Treg family includes natural Tregs(nTregs) that express the nuclear transcription factor FoxP3, as well as cell surface proteins CTLA-4 and CD25 and induced Tregs(iTregs) [8, 9]. An increase of Tregs in fibroproliferative sites influences the subsequent balance between tissue inhibitors of metalloproteinase(TIMP) and matrix metalloproteinase(MMP) and Kupffer cell (KC) expression to promote ECM remodelling and fibrogenesis [10]. In addition, the Treg/TH17 ratio plays a key role in the process of intrahepatic immune regulation and the dysregulation of this ratio is a key characteristic of the progression of liver fibrosis [7]. Restoring Treg/Th17 balance can effectively immunize against various intracorporal and extracorporeal pathogens, as well as prevent excessive autoimmune self-harm [11]. However, further research on Tregs and the Treg/Th17 role in the fibrogenesis interactome is needed to promote potential diagnostic and therapeutic options. This review outlines the current understanding of the role/s of Tregs on liver fibrosis. First, we review the known pathways of Tregs that promote fibrogenesis and second the role of Tregs and their related cytokines that suppress fibrogenesis. Thirdly, we outline the potential effects of the Treg/Th17 balance and their related cytokines on fibrogenesis and finally, we illustrate the complex Treg interactome and discuss the potential for Treg therapy to be exploited for the treatment of fibrosis of the liver.

## Background and role of Tregs

Treg cells were first discovered by GERSHON et al. [12] in the 1970s and were referred to as “suppressor cells” because of their role in immune regulation. A later study in 1995 showed that CD25 expression was a hallmark of these suppressor cells that were labelled for the first time as Tregs [13]. This study also demonstrated that the in vivo transfer of CD4^+^CD25^+^ Treg-deficient T cells triggered autoimmune disease in a mouse model. Conversely, autoimmune effects were retarded when CD4^+^ CD25^+^ cells were introduced. The prevailing view is that there are two main sub-types of Tregs determined by cellular origin, namely, natural Tregs(nTregs), which are differentiated in the thymus from progenitor cells derived from bone marrow, and inducible Tregs (iTregs), which are differentiated from peripheral naïve T cells to respond to infectious challenges. Except for nTregs and iTregs, various studies have identified other T cells with regulatory properties, including T helper (Th)-like Treg subsets, CD8^+^Tregs, Tr1 cells, and Th3 cells [14–17]. In addition, many researchers have subdivided Treg cells into different subsets in different diseases according to the different expression of Treg cell surface molecules, such as “Treg B” that shows a higher expression of CD95, CCR4, and CD45RO within FOXP3^hi^, CD127^lo^ Tregs in aplastic anaemia [18]. Transcription factor forkhead box protein 3 (FoxP3) is the most characteristic marker of Treg cells and plays an important role in Treg development and immune regulation. Tregs mainly secrete IL-10, TGF-β, IL-35, and other cytokines, which enable the body to obtain immune tolerance by inhibiting the differentiation and proliferation of NKT cells, macrophages, B cells, and so on [19]. Treg cells express the following molecules on their surface: CD25, CTLA-4, PD-L1, GITR, and HLA-DR [20, 21]. The expression and functions have significant differences between naïve and activated Tregs, suggesting another dimension that the tissue Tregs express higher levels of activation markers compared to blood. For example, activated effector Tregs (CD45RA-FOXP3high) express high levels of HLA-DR compared to naïve Tregs (CD45RA^+^FOXP3low) [22, 23] and activated Treg cells play a major role in fibrogenesis. Although Treg cells only account for 5%-10% of the total CD4^+^ T lymphocytes in peripheral blood [24], their contribution to maintaining immune tolerance and homoeostasis cannot be ignored.

## The pro-fibrosis function of Treg

**Fig. 1:**
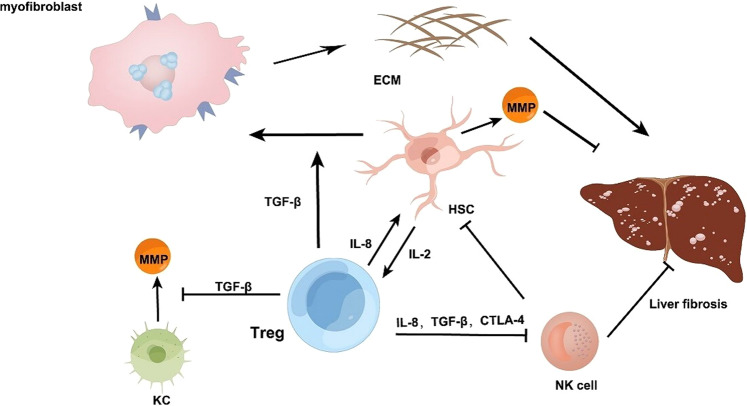
The role of Treg in promoting fibrosis. Liver fibrosis is primarily induced by HSCs that promote TGF-β based production of myofibroblasts that stimulate ECM-driven fibrogenesis. Tregs can also activate HSCs to promote fibrosis through the TGF-β pathway. Treg can induce HSCs via the expression of IL-8. Treg cells with CD4^+^CD25^+^Foxp3^+^ can be selectively expanded by HSCs in an IL-2–dependent pattern. Tregs can directly suppress NK cells from degranulating in the role of IL-8, TGF-β1, and CTLA-4 signalling pathways. Footnote: hepatic stellate cells (HSCs), transforming growth factor-β (TGF-β), extracellular matrix (ECM), matrix metalloproteinase (MMP), regulatory cells (Treg), kupffer call (KC), interleukin-2 (IL-2), natural killer cell (NK cell), cytotoxic T-lymphocyte-associated protein 4 (CTLA-4).

Activated Tregs are essential during the healing period to end inflammation and promote wound healing [25] and yet recent evidence indicates that a large number of Tregs are distributed in the fibrotic microenvironment in HCC patients. Interestingly, a reduction in Tregs promoted the regression of fibrosis [26] illustrating that Tregs play a prominent role in promoting fibrosis [Fig. 1]. A report showed Treg cells with CD4^+^CD25^+^Foxp3^+^ were selectively increased by HSCs in an interleukin-2–dependent pattern [27] explaining the abundance of Tregs in fibrotic tissue [Fig. 1]. MMPs, the main enzyme involved in ECM degradation [28] are produced primarily by KCs [29], however, even a low ratio of Tregs can repress KC expression of MMPs. Treg cells regulate the balance of MMP/TIMP through KCs, thereby inhibiting the resolution of fibrosis [4] [Fig. 1].

Experiments have shown that Tregs may inhibit KCs from secreting MMP in vivo through the TGF-β pathway [30] [Fig. 1]. A parallel increase in collagen type I deposition and TGF-β1^+^Tre g cells was observed in lymphoid tissues of the simian immunodeficiency virus (SIV) -infected rhesus monkeys 7 days after inoculation [31]. These results indicate that TGF-β1^+^ Treg cells have a dichotomous response to immune activation; on the one hand, they can inhibit viral immune response while on the other hand, they can induce collagen deposition in lymphoid tissues, resulting in organ fibrosis.

HSCs which are spread over the Disse space are generally considered to be the hallmark cells of fibrosis and the major source of myofibroblasts and activation of HSCs is the initiating event of hepatic fibrosis. HSCs themselves can regulate the development of fibrosis. On the one hand, in the presence of inflammatory stimuli, resting HSCs are often activated and converted into myofibroblast-like cells and generate large amounts of ECM and cytokines [Fig. 1]. On the other hand, activated HSCs can secrete MMPs to degrade ECM [32] [Fig. 1]. It can be said that HSCs largely determine the promotion or regression of liver fibrosis. In addition, TGF-β1 secreted by macrophages is the strongest known activator of HSCs [33]. Meanwhile, the two most important cytokines secreted by Tregs are TGF-β and IL-10 [34] and Tregs can activate HSCs to promote fibrosis through the TGF-β pathway [Fig. 1].

Of note, HBV which is one of the main pathogenic factors leading to liver fibrosis can induce the generation of immunosuppressive cells, such as Tregs, MDSCs, and NK-REG cells, through the immunosuppressive cascade, undue induction can lead to adverse outcomes, such as liver fibrosis and even HCC [35]. HBV-induced Tregs may suppress the antifibrotic function of NK cells and directly suppress NK cells from degranulating in the role of IL-8, TGF-β1, and CTLA-4 signalling pathways [Fig. 1]. Tregs can also have an indirect protective effect on HSCs from NK cell’s attack on account of suppressing the expression of MICA/B on HSCs through TGF-β1 and IL-8 [36]. Tregs can inhibit NK cells and M1 KCs from participating in the immune regulation of liver fibrosis, and its immunosuppressive regulation contributes to the development of chronic inflammation, thus maintaining liver fibrosis [37]. Therefore, by cutting off the pathway of Treg action on NK cells and restoring antiviral T-cell responses, the development of inflammation and injury can be limited, and HSCs, as a crucial element of liver fibrosis, can be reduced, thereby limiting the development of liver fibrosis.

In addition, according to experimental observation, Treg cells have been shown to proliferate during chronic HCV infection [38] and are enriched in liver fibrosis tissue to protect HSCs from NK cell attack [36]. Interestingly, an abundance of T cells with IL-8 ^+^CD4^+^Foxp3^+^ are found in HCV liver tissue (mainly in fibrotic and α-SMA regions), it has also been demonstrated that deletion of IL-8 can inhibit the activation of HSCs, however, the immune regulatory function of Treg cells was not affected, illustrating that IL-8^+^ Tregs can promote fibrosis [39] [Fig. 1].

In summary, the complex Treg interactome reveals that Treg expression can secrete IL-8 to activate HSCs that, in turn, can secrete IL-2 to induce Treg expression. Tregs can secrete TGF- β to repress KC expression of MMPs that degrade ECM accumulation to promote fibrogenesis and Treg-secreted TGF- β promotes HSC-derived myofibroblasts. Tregs also promote fibrogenesis by secreting Il-8, TGF- β and CTLA-4 to repress NK repression of HSC activation. Tregs thus interact with multiple cell types and activate a range of cell–cell interactions with HSCs, NKs and KCs to promote fibrogenesis (Fig. 1).

## The anti-fibrosis function of Treg

**Fig. 2:**
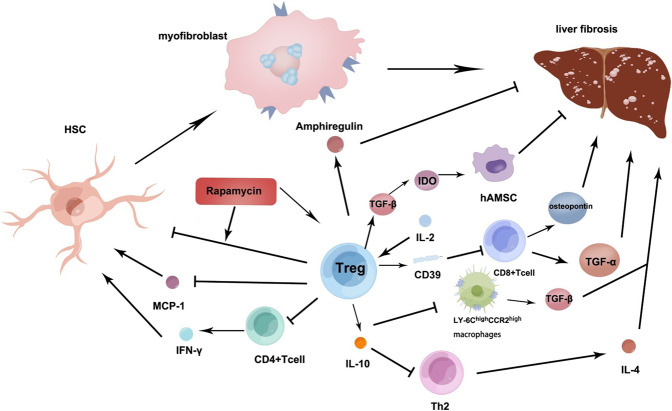
The role of Treg in inhibiting fibrosis. IL-2 as well as its complexes accelerate Tregs expressing CD39 in the liver, thereby inhibiting the multiplication of CD8^+^ T cells and its function of generating TNF-α and osteopontin, which can reduce biliary fibrosis. Tregs can regulate the pro-fibrotic roles of Th2 cells and Ly-6C^high^CCR2^high^ inflammatory monocytes/macrophages that secrete IL-4 and TGF-β respectively, interestingly, IL-10 secreted by Tregs may regulate them. Tregs can regulate the TGF-β-IDO signalling pathway to enhance the function of hAMSC that repress liver fibrosis. Tregs promoting the expression of amphiregulin can inhibit the development of fibrosis by promoting the proliferation of hepatocytes. In CCL4-induced liver fibrosis, rapamycin has an effective protective effect on the liver. Rapamycin significantly increased the functional activity of CD4^+^CD25^+^ Tregs and enhanced the inhibitory ability of Tregs on HSCs activation. Tregs can repress MCP-1 which plays an important role in liver fibrosis by activating HSC production. Tregs can directly repress CD4^+^ T cell expression that reduces IFN-γ activated HSCs. Tregs also directly promote amphiregulin to repress liver fibrosis. Footnote: interleukin (IL), regulatory cells (Treg), transforming growth factor-α (TGF-α), T helper cell (Th), indoleamine 2,3-dioxygenase (IDO), human amniotic mesenchymal stromal cell (hAMSC), carbon tetrachloride(CCL4), hepatic stellate cells (HSCs), monocyte chemotactic protein-1 (MCP-1), Interferon-γ (IFN-γ).

It is interesting to note that TGF-β generated by Tregs in HCV is inversely associated with the regression of liver inflammation and fibrosis, even though cytokine TGF-β is a well-established pro-fibrotic element, suggesting that TGF-β also has antifibrotic properties [40]. According to the observation, the large number of highly differentiated and activated Tregs distributed to infiltrating chronic HCV-infected livers can lead to a limitation of the degree of fibrosis. [41] Tregs in the peripheral blood of HCV patients can suppress the proliferation of HCV-specific T cells and IFN-γ [42]. In addition, the depletion of Tregs restored the secretion of IFN-γ by CD4^+^ T cells [43] [Fig. 2]. This indicates that Tregs in an HCV-induced environment are pivotal in the limitation of collateral injury and homoeostasis maintenance by inhibiting an intemperate immune response.

Treg cells can guard against HIV-1-induced liver fibrosis, and the mechanism may be related to the activation of HSCs, liver injury, and hepatitis [44]. In this regard, one study investigating HIV-1-induced liver fibrosis deleted Treg cells with denileukin diftitox. By measuring various plasma indicators, Only HIV-1+ denileukin diftitox mice in infection showed a significant increase in ALT levels after 20 days. Similarly, serum hyaluronic acid levels, also a sign of liver injury and fibrosis, were only elevated in infected HIV-1+ denileukin diftitox mice after 20 days. Therefore, HIV-1 infection together with Tregs depletion causes liver inflammation and ultimate fibrosis. Using Denileukin Diftitox alone highly up-regulated the expression of human inflammatory chemokines McP-1 and MIP-1α in the liver [Fig. 2]. It revealed that McP-1, which is closely related to Tregs, may play an important role in the process of fibrosis initiated by inflammation [45].

While protecting the body from physiological factors, inflammatory cell immunity also promotes liver injury and fibrosis. Tregs can inhibit inflammatory cell immunity, thereby inhibiting fibrosis. It has been found that chronic liver inflammation induced by carbon tetrachloride (CCL4) injection tends to preferentially expand liver Treg cells, thereby protecting liver function and avoiding fibrosis [46]. In CCL4-induced liver fibrosis, rapamycin has an effective protective effect on the liver. Further studies found that rapamycin significantly increased the functional activity of CD4^+^CD25^+^ Treg, and enhanced the inhibitory ability of Tregs on HSCs activation [47] [Fig. 2]. α-SMA is a hallmark of the activation of HSCs and a sensitive indicator of myofibroblasts [48]. It was also found that increased Treg cell levels, induced by rapamycin, effectively decreased α-SMA expression [47]. Alternatively, Treg depletion enhances the immunologic function of inflammatory cells and drives pro-fibrotic T-helper 2 cells(Th2) that induce IL-4 activation. Th1 cells, which secrete IFN-γ and Th2 cells which generate IL-4, are antifibrotic and pro-fibrotic T-helper cell subsets, respectively. Treg depletion will break the balance between these two subsets, thus leading to fibrosis [49]. Th2 cells and Ly-6C^high^CCR2^high^ inflammatory monocytes/macrophages both have a pro-fibrotic function by secreting IL-4 and TGF-β respectively, although IL-10 secreted by Tregs may regulate them [50] [Fig. 2]. This illustrates that although Tregs can promote fibrosis, most evidence suggests that Treg cells have an anti-fibrotic effect, primarily due to their immunosuppressive effect on IL-10 secretion [51]. In addition, Tregs also secrete amphiregulin, an important factor involved in tissue repair and regeneration in a variety of models of inflammation [52]. In one study, amphiregulin inhibited the development of fibrosis by promoting the proliferation of hepatocytes [53] [Fig. 2].

In a model of acute disease induced by bile duct ligation(BDL), Tregs in the liver reduced T lymphocyte function and effectively limited liver fibrosis [54] demonstrating the critical role of Tregs in maintaining immune suppression. Furthermore, in the BDL model, the exhaustion of Tregs increased the generation of inflammatory mediators, such as chemokines and cytokines, and promoted the infiltration of Th17 and CD8^+^ T cells in the fibrotic liver [55], suggesting that Tregs can suppress the pro-fibrotic effects of Th17 and CD8^+^ T cells. In a mouse model with sclerosing cholangitis, treatment with low-dose IL-2 induced the expansion of intrahepatic Tregs, thereby reducing biliary injury and fibrosis. The mechanism may be Il-2 precipitated, as well as its complexes, that promote Tregs expressing CD39 in the liver, thereby inhibiting the multiplication of CD8^+^ T cells and its function of generating TNF-α and osteopontin, which can reduce biliary fibrosis [56] [Fig. 2].

Human amniotic mesenchymal stromal cells (hAMSC) can regulate the immune response in a variety of diseases and demonstrate a powerful regenerative repair ability similar to stem cells [57]. The combination of Treg and hAMSC infusion rescued low-grade liver fibrosis compared with injecting Treg or hAMSC respectively. The reason may be that Tregs can improve the expression of hepatocyte growth factor (HGF) and the ability of hAMSC to differentiate [58] [Fig. 2]. The two principal cytokines expressed by Tregs are TGF-β and IL-10 [34, 59]. Experiments have shown that Tregs can regulate the TGF-β-IDO signalling pathway and then enhance the function of hAMSC, and combination therapy of Tregs and hAMSC infusion has great potential research value for the treatment of liver cirrhosis [58] [Fig. 2]. It was also proved that mesenchymal stem cells(MSCs) as well as MSC-conditioned medium (MSC-CM) both inhibited necroinflammatory and fibrogenesis in a chronic liver injury model, however, the second therapy had a better effect. In this regard, the activation of Treg and Th2 cells, and decreased the number of Th17 cells [60]. Another study showed that umbilical cord-derived MSCs selected by individual heterogeneity have the function of promoting Tregs and have been proven to improve the recovery of liver fibrosis in mice [61]. Tregs can also alleviate the pathological process of hepatic steatosis and high-calibre abnormal blood cholesterol and glucose metabolism, and aberrant levels of liver enzymes in leptin-deficient OB/OB mice [62], indicating the potential curative effect of diabetes and prevention of early hepatic fibrosis by inducing Tregs.

In summary, the complex Treg interactome that induces an anti-fibrotic effect involves the interaction between Tregs and a range of liver cell types and signalling pathways. Rapamycin can effectively protect the liver by enhancing the inhibitory ability of Treg to the activation of HSCs. Tregs repress HSC activation by repressing MCP-1-induced HSC activation and by repressing CD4^+^ T-cell secretion of IFN-γ. This anti-fibrotic interactome also includes repression of Th2/IL-4 driven fibrosis, the repression of macrophage/TGF-β initiated fibrosis, and the promotion of hAMSC repression of fibrosis by promotion of TGF-β/IDO induced hAMSC. Tregs repress fibrosis by inducing amphiregulin repression of fibrosis and Tregs can induce IL-2/CD39 repression of CD8^+^ T-cells to suppress fibrosis because they reduce CD8^+^ T-cell expression of TGF-α and osteopontin. It is, therefore, apparent that Tregs interact with multiple cell types in multiple pathways to repress liver fibrosis (Fig. 2).

## The Treg/Th17 balance in liver fibrosis

**Fig. 3:**
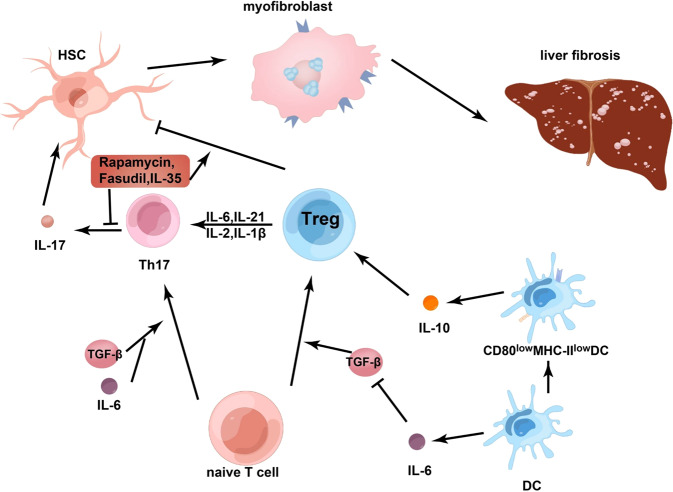
The relationship between Tregs and Th17. Treg and Th17 cells are convertible in different immune microenvironments. Treg cells can be transformed into cells expressing IL-17 in the presence of IL-6 and IL-21, as well as IL-2 and IL-1β. Fasudil can effectively inhibit cell differentiation of Th17 and its ability to express IL-17. Using this inhibitor and upregulation of Treg can limit liver fibrosis in mice caused by Schistosoma japonicum infection. Rapamycin significantly reduced the level of Th17 cells in the CCL4-induced liver fibrosis model, it even can enhance the inhibitory function of Treg on HSCs.IL-35 has a similar function compared with the formers. Il-6 has an extraordinary effect on Th17/Treg balance. Il-6 can induce the development of naïve T cells into Th17 cells in the existence of TGF-β. However, its effect on Tregs has two sides. But overall, IL-6 still tilts the balance between Tregs and Th17 toward Th17 cells. DCs play dual roles in the pathways of Treg. Footnote: regulatory cells (Treg), T helper cell (Th), interleukin (IL), retinoid-related orphan receptor gamma t (RORγt), carbon tetrachloride (CCL4), hepatic stellate cells (HSCs), dendritic cells (DC).

Th17 cells are a subpopulation of CD4^+^ T cells that specifically express RORγt and secrete signature cytokines IL-17, IL-22, and IL-23 [63]. Recent studies have found that Th17 and the cytokines it secretes have a significant impact on the pathological process of liver fibrosis [64–66].Th17 cells are bound up with Treg cells. First, both Th17 and Treg cells are derived from naive CD4^+^ T cells, and second, they both require TGF-β signalling to differentiate. Interestingly, the two cells that are so similar have diametrically contrary functions: Th17 cells contribute to the development of inflammation and autoimmunity, while Treg cells suppress the above-mentioned phenomena and promote immune homoeostasis [67] [Fig. 3]. In vitro HSCs can be activated by Th17 to develop fibrosis while Tregs can inhibit the activation of HSCs [11].

Some viruses can persistently damage liver tissue, eventually leading to cirrhosis, and Treg/Th17 balance can prevent excessive liver damage during viral infection. Some studies have found that in acute hepatitis A virus(HAV) patients, serum IL-17 level has a positive correlation with the degree of liver damage, and the frequency of hepatic resident and circulating Tregs is negatively correlated with liver injury [68]. Similarly, other researchers have demonstrated that the quantity and proportion of Th17 cells in the liver and peripheral blood are increased in acute and chronic liver injury [69, 70] demonstrating the strong pro-fibrotic function of Th17.

It has been found that a selective Rho/RhoA-associated kinase (ROCK) inhibitor (Fasudil) can effectively inhibit cell differentiation of Th17 and its ability to express IL-17 [Fig. 3]. Using this inhibitor and the upregulation of Tregs can limit liver fibrosis in mice caused by Schistosoma japonicum infection [71]. Treg/Th17 play opposite roles in the development of HBV-LF fibrosis by affecting liver injury and hematopoietic stem cell function [72]. This is demonstrated, not only in liver fibrosis, but also proved that Treg/Th17 balance exists in several inflammatory and autoimmune diseases, including systemic lupus erythematosus (SLE), rheumatoid arthritis, and primary biliary cirrhosis, [73–75]. Therefore, we deduce that the Treg/Th17 balance may be an important biomarker of immune homoeostasis, reflecting some extent the balance between the pro-inflammatory and anti-inflammatory abilities of the body.

The balance of Treg /Th17 is so important in the progression of cirrhosis that some scholars have considered it an independent predictor of decompensated cirrhosis [72]. It has also been suggested that the Treg/Th17 ratio may be used as a measure of disease severity in animal models and human diseases [76]. Zhai et al. showed that the ratio of Th17 to Treg had a negative correlation with the survival rate of patients with acute-on-chronic liver failure(ACLF) [77]. The experimental results of Yu et al. also showed a similar conclusion, and they found that the ratio of Treg to Th17 in the survival group was higher than the ratio in the non-survival group of HBV-LC patients [78]. Nan et al. observed that compared with the control group, the cell quantities of Treg and Th17 cells in the chronic HBV patients’ peripheral blood were larger, and the proliferation of Th17 cells was more obvious than that of Treg cells in the acute attack, and this series of changes led to the decrease of Treg/Th17 ratio [79]. Rong et al. reported that the number of Th17 cells and the level of ROR-γT expression in the peripheral blood of patients suffering from primary biliary cirrhosis were significantly increased. However, the quantity of Treg cells and the expression of FoxP3 were significantly decreased [73]. Another study demonstrated that a lower ratio of Treg to Th17 often indicates greater progression of liver fibrosis in HBV-infected patients [40]. These results suggest that the imbalance between Th17 and Treg is strongly associated with the development of liver cirrhosis, and the balance of them may be a key prognostic indicator for HBV-LC, and an increased Treg/Th17 ratio may indicate a better prognosis, whereas a poor prognosis otherwise.

In addition, lL-35 has a crucial impact on balancing Treg and Th17 in acute and chronic HBV infection. It has been found to amplify the functions of virus-specific Treg cells while also inhibiting Th17 cell differentiation [80] [Fig. 3]. It is this effect of IL-35 that could lead to the break of balance, which causes persistent HBV infection and chronic disease, ultimately leading to irreversible liver fibrosis. It was also found that rapamycin significantly reduced the level of Th17 cells and the expression level of ROR-γT in the CCL4-induced liver fibrosis model. At the same time, rapamycin can lead to a significant increase in Treg frequency and Foxp3 expression and it can even enhance the inhibitory function of Tregs on HSCs [47] [Fig. 3]. From the different effects of rapamycin on Treg and Th17 expression, as well as its inhibition of the development of fibrosis, rapamycin appears to play an important role in the regulation of Th17/Treg balance.

As mentioned above, Tregs play different roles under different cytokine conditions. However, increasing data indicate that Treg and Th17 cells are convertible in different immune microenvironments. For instance, Treg cells can be transformed into cells expressing IL-17 in the presence of IL-6 and IL-21 [Fig. 3], while Th17 cells can be transformed into Th1/2 cells in the existence of IL-4 or IL-12 [81]. Deknuydt et al found that nTregs can be transformed into Th17 cells under the induction of IL-2 in the immune microenvironment where the activated APC and monocytes after microbial stimulation are considered to be the most effective factors in promoting Treg transformation [Fig. 3]. Further studies showed that IL-1β could mediate the conversion of Tregs to Th17 [Fig. 3], which may be related to the downregulation of Foxp3 [82]. Over the last few decades, the main research directions on liver fibrosis are Th1 and Th2-focused. Recent advances in immunology have focused on two new T cell subsets (Treg and Th17 cells) that supplement and refine the classical theory of Th1/Th2 [83] interplay. For example, Il-6 has a significant effect on Th17/Treg balance [84] and can induce the development of naïve T cells into Th17 cells in the presence of TGF-β. On the contrary, IL-6 has both direct and indirect effects on Treg cells. On the one hand, IL-6 suppressed the induction of Treg cell conversion by TGF-β without affecting the number and function of nTregs [85, 86] [Fig. 3].On the other hand, there are several lines of evidence that IL-6 has immunomodulatory effects supporting Tregs number and function, which are mainly because LPS-stimulated dendritic cells produce IL-6 which activates STAT3 signalling to eventually keep DCs in an immature state with low CD80 and MHC-II levels, as well as increase IL-10 production to promote Treg cells [87] [Fig. 3]. But overall, IL-6 still tilts the balance between Tregs and Th17 toward Th17 cells.

In summary, Th17 and Treg cells are integrally involved in the immune response in the progression of liver fibrosis. Their balance determines the maintenance of body homoeostasis and is very important for the prevention or prognosis of liver fibrosis. The complex Treg interactome that results in the differentiation of naïve T-cells influencing the Treg/Th17ratio and its influence on fibrogenesis involves the interaction of a differential Treg exposure to a range of cytokines, as well as cell-to-cell signalling pathways. Tregs exposure to IL-6/-2/-21/-IL-1β can be transformed into Th17 to promote HSC initiate firogenesis. Naïve T-cells differentiation to Th17 promotes IL-17-promoted HSC activation. On the other hand, rapamycin/fasudil/Il-35 expression can promote Treg to inhibit HSC activity and inhibit Th17 to promote HSC activation. DCs can secrete IL-6/ TGF-β that repress naïve T-cell differentiation to Tregs while TGF-β promotes Th17 differentiation (see Fig. 3).

## Conclusion

The mechanism of Tregs in liver fibrosis has not been completely elucidated, Whether Tregs express pathogenicity or host protective functions often depends on environmental stimuli. Because of the duality of Tregs in different environments, this area of research has become a ‘hotspot’ in recent years. This is important because the role of Tregs is involved in several risk factors contributing to liver fibrosis, such as hepatitis virus, schistosomiasis, alcohol, certain drugs, toxicants and NCDs. In summary, liver fibrosis is mainly divided into two aspects, one is the damage of hepatocytes or cholangiocytes, and the other is the disorder of ECM production. In response to environmental cues for fibre resolution or fibre formation, Tregs show different transcriptional changes. We can conclude that the transcriptional capacity of Tregs is tissue-specific and can be expressed differently in response to different environmental or influencing factors. Moreover, the role of Tregs can even express reverse effects at different stages of the disease. This is aptly demonstrated by research that shows that Treg cells were depleted early in bleomycin-induced fibrosis by anti-CD25 antibody and the prognosis of fibrosis was improved, however, when Treg cells were depleted late in the disease, fibrosis was exacerbated [88]. Besides, the role of Tregs in the same disease often has two sides, the function of Tregs in the pathological process of hepatitis B is contradictory and complex. On the one hand, Tregs can inhibit the promoters of liver fibrosis (HSCs), yet at the same time, this can provide a haven for the hepatitis B virus to avoid immune detection [89].

Studies over the past decade have shown that Th17 cells have a proinflammatory response in almost all tissues [90]. The effect of Tregs is not always opposite to Th17, instead, under certain conditions, the two can act synergistically. The Treg/Th17 relationship may also be related to different irritants, triggers of liver injury, stages of fibrosis, and interactions between different immune mediators. Due to the dichotomous nature of Tregs, perhaps targeting liver fibrosis by immune cells can open a new path for patients. Furthermore, Treg/Th17 interaction may help us analyze the disease status, judge the prognosis of patients and promote diagnostic and therapeutic options to manage liver fibrosis. Future research is needed to understand the mechanism and balance of Treg and Th17 cells in liver tissue, as well as investigate different signalling pathways that may affect Th17/Tregs’ contribution to liver-specific therapies. Finally, we have attempted to highlight the complex Treg interactome in its promotion of fibrosis, in its alternate anti-fibrotic role and lastly how the Treg/Th17 ratio influences pro and anti-fibrotic activity. The multiplicity of interacting cells with Tregs in the liver, as well as the signalling pathways suggest that multiple therapeutic options can be further explored, however, a fuller understanding of liver fibrogenesis will be needed.

## Data Availability

All data included in this study are available upon request by contact with the corresponding author.
